# Autoimmune Disease-Associated Hypertension

**DOI:** 10.1007/s11906-019-0914-2

**Published:** 2019-02-02

**Authors:** Victoria L. Wolf, Michael J. Ryan

**Affiliations:** 1 Department of Physiology & Biophysics, University of Mississippi Medical Center, 2500 North State Street, Jackson, MS 39216-4505 USA; 2 G.V. (Sonny) Montgomery Veterans Affairs Medical Center, Jackson, USA

**Keywords:** Autoimmunity, Hypertension, Lupus, Inflammation, Renal hemodynamics, Vascular function

## Abstract

**Purpose of Review:**

To highlight important new findings on the topic of autoimmune disease-associated hypertension.

**Recent Findings:**

Autoimmune diseases including systemic lupus erythematosus and rheumatoid arthritis are associated with an increased risk for hypertension and cardiovascular disease. A complex interaction among genetic, environmental, hormonal, and metabolic factors contribute to autoimmune disease susceptibility while promoting chronic inflammation that can lead to alterations in blood pressure. Recent studies emphasize an important mechanistic role for autoantibodies in autoimmune disease-associated hypertension. Moving forward, understanding how sex hormones, neutrophils, and mitochondrial dysfunction contribute to hypertension in autoimmune disease will be important.

**Summary:**

This review examines the prevalent hypertension in autoimmune disease with a focus on the impact of immune system dysfunction on vascular dysfunction and renal hemodynamics as primary mediators with oxidative stress as a main contributor.

## Introduction

Autoimmune disease is a major global health burden that affects approximately 5% of the population. For reasons that remain unclear, the prevalence of autoimmune diseases such as psoriasis, rheumatoid arthritis (RA), and systemic lupus erythematosus (SLE) has been increasing [1–3]. Cardiovascular disease is the leading cause of mortality, and its prevalence is markedly increased in patients with autoimmune diseases [4]. Hypertension is a major modifiable cardiovascular disease risk factor that is also prevalent in patients with autoimmune diseases [5, 6]. Despite the prevalent hypertension, guidelines for the management of hypertension do not consider patients with autoimmune disorders like SLE, causing practitioners to rely on the existing recommendations for the general population while lacking data from large-scale clinical trials [7•]. Although anti-hypertensive medications are commonly indicated for patients with autoimmune disease, many patients are not prescribed the appropriate therapy, and those who are taking anti-hypertensive medications often have difficulty achieving guideline-recommended treatment targets [8]. Blood pressure is controlled by a complex, integrative network of physiological systems that involves renal, neurological, endocrine, and vascular mechanisms. Work from our laboratory and others suggests that innate and adaptive immunity are important regulators of these physiological systems and therefore have important mechanistic implications for the development of hypertension [9•, 10–12]. The purpose of this review is to highlight recent insights into how the chronic inflammation associated with autoimmunity may contribute to hypertension. Although multiple autoimmune diseases have prevalent hypertension and will be discussed herein, the major emphasis of this review will be on SLE, as a disease model of autoimmune-associated hypertension. More specifically, the review will focus on vascular dysfunction, renal hemodynamic mechanisms, and the role of oxidative stress. Several comprehensive reviews of the role that immunity has in the pathogenesis of hypertension are already available [13–15]. In addition, factors that may potentially serve as permissive mediators of autoimmune disease-associated hypertension will be discussed.

### Hypertension Is Prevalent in Patients with Autoimmune Disease

Clinical evidence shows that there is a strong association between autoimmune diseases like SLE and RA with hypertension [16]. For example, a large population-based study found an increased prevalence of hypertension in patients with RA (31%) compared to the general population at 23% [17]. Several studies show an increased prevalence of hypertension in patients with SLE reaching as high as 40% of SLE patients under the age of 40 [18–20]. Similarly, patients with scleroderma have prevalent hypertension, especially when there is renal involvement [21]. Autoimmune disorders including SLE, RA, and scleroderma occur after a loss of immune tolerance with the subsequent production of autoantibodies. Interestingly, autoantibodies are associated with hypertension in patients with SLE, and primary hypertension is associated with an increase in serum immunoglobulins and increased antinuclear antibodies [22]. The presence of autoantibodies in patients with primary hypertension offers clues about the possible autoimmune underpinnings of the disease; however, we are just now beginning to understanding the link between autoimmunity and hypertension.

### Blood Pressure Control in Patients with Chronic Autoimmune Disease

Despite an increased prevalence of hypertension and corresponding increase in cardiovascular risk, hypertension treatment guidelines do not consider the potential needs or challenges that might be unique to patients with autoimmune diseases like SLE, and drugs commonly used in the treatment of SLE have the potential to impact blood pressure [7•]. For example long-term use of glucocorticoids, non-selective NSAIDS and cyclooxygenase II inhibitors (coxibs), and some disease-modifying antirheumatic drugs (DMARD) are all associated with an increased risk for hypertension [16]. Part of the difficulty controlling blood pressure in patients with autoimmune disease may also be related to the prominent renal disease in patients with SLE. Approximately 40–70% of patients with SLE will develop chronic kidney disease (CKD) [23], and while upwards of 80% of patients with CKD have hypertension [24], only 13% have adequately controlled blood pressure [25]. Although no randomized-controlled trials have been performed, angiotensin converting enzyme (ACE) inhibitors are commonly prescribed for the treatment of hypertension and/or renal disease in SLE patients. The use of ACE inhibitors during SLE is generally well tolerated and associated with a delay in the onset of renal involvement and a decline in the risk of disease relapse in SLE patients [26] that likely occurs from both the decrease in angiotensin II and the immunomodulatory impact of renin-angiotensin system blockade. The premature development of atherosclerosis is less frequent among SLE patients using ACE inhibitors [27]. Nevertheless, the appropriate initiating drug for blood pressure control, combinations of drugs, and the potential impact of immunomodulatory therapies on blood pressure in this patient population has not been carefully examined.

### Risk Factors for Autoimmune Disease and Hypertension

Both SLE and hypertension are multifactorial diseases. Therefore, it may not be surprising that there is overlap among genetic and environmental risk factors for the development of autoimmune diseases and hypertension. For example, smoking and body mass index (BMI) associated with poor outcomes for patients with SLE and with the risk of developing hypertension [6, 28–30]. However, patients with SLE have an increased risk for CVD mortality beyond traditional risk factors, suggesting that disease-specific factors play an important role [31]. Below, some of the SLE-specific risk factors, which contribute to SLE susceptibility and likely contribute to the increased prevalence of hypertension, are discussed.

### Genetic Factors

Numerous susceptibility genes have been identified in the pathogenesis of autoimmune diseases. Studies in the NZM2410 model of SLE in 1994 by Morel et al. led to the identification of three susceptibility loci, Sle1-3, that have a significant correlation with the development of immune-complex-mediated glomerulonephritis in murine strains [32]. Studies have been done to link insertion/deletion polymorphisms in the gene for ACE to SLE, lupus nephritis, and vascular changes; although, no strong correlation has been identified [33–35]. Similar studies have shown no definitive correlations in patients with systemic sclerosis [36]. HLADR4, DRβ1, protein tyrosine phosphatase 22, and peptidyl arginine deiminase 4 (PADI4) are among the genes identified with an association for an increased susceptibility in patients with RA [37]. One recent study also suggests an association with PADI4 gene polymorphisms and patients with SLE with additional PADI4 polymorphisms conferring an increased risk of renal involvement in the form of lupus nephritis [38]. PADI4 is an enzyme responsible for the conversion of arginine to citrulline residues, which is an important step in the process of NETosis. NETosis will be discussed in more detail later on as it pertains to the formation of oxidative stress. Although these susceptibility genes have a known association with immune system dysfunction in autoimmunity, it is not clear how or if these susceptibility genes contribute to the development of hypertension.

### Environmental Factors

Environment/gene interactions that increase autoimmune disease susceptibility have been studied for many years and the understanding of these interactions continues to evolve [28, 39]. Recently, vitamin D deficiency has been reported in many patients with autoimmune disease [40–42]. Vitamin D deficiency in patients with SLE is linked to prevalent renal disease, in addition to lifestyle modifications (sunlight avoidance due to photosensitivity) and medications (long-term use of corticosteroids) [43]. Potential mechanisms by which vitamin D deficiency may contribute to the pathogenesis of SLE include an associated decrease in regulatory T cells (T_REG_), which are essential for immune tolerance against self-antigens, and increased activation of autoreactive B cells, contributing to an increased generation of autoantibodies [44]. The immunomodulatory properties of vitamin D are mediated by vitamin D_3_ receptors (VDR) found on multiple immune cell lineages (monocytes, dendritic cells, and activated T cells) [45]. In addition to the known link between vitamin D deficiency and immune system activation, there is evidence for an association between vitamin D deficiency and an increased risk for hypertension and cardiovascular disease [43]. A recent study by Sabio et al. reported that there is a strong association between non-dipper hypertension and vitamin D deficiency in women with SLE [46]. Non-dipper hypertension is defined as the absence of a normal 10% physiological decline in blood pressure that occurs at night, and is linked with a greater risk of developing cardiovascular disease (as summarized in this review [47]). Going forward, it will be important to continue identifying and understanding the mechanisms by which environmental factors like vitamin D deficiency can modulate autoimmunity and the associated risk for developing hypertension.

### Sex-Specific Factors

The idea that the immune system can impact blood pressure in a sex-dependent manner is supported by numerous studies (summarized in these recent reviews [48–50]). Sex hormones, including estrogen and testosterone, have immunomodulatory functions and are often implicated in the strong female sex bias associated with autoimmune diseases like SLE and RA. In experimental models of autoimmunity, the manipulation of sex hormones has been shown to modify the pathogenesis and progression of disease. Estrogen (via ERα) has long been the primary focus of researchers trying to understand the sex bias in SLE; however, a recent study challenged the notion that complete knockout of ERα is directly protective in murine lupus [51•]. Scott et al. demonstrated that NZM/ERαKO mice were not protected from renal disease following ovariectomy [51•], a finding that suggests other ovarian hormones might confer protection in the absence of ERα. Continuing to understand the role of estrogens during SLE and the potential regulation of blood pressure in these women will be an important pursuit. Estrogens are typically thought to confer protection against cardiovascular risk factors like hypertension because women have a lower prevalence of hypertension than men until they reach menopause [52]. The complex role of estrogens during SLE was further illustrated by work from our own laboratory showing that ovariectomy in adult female mice with SLE (30-week old NZBWF1) exacerbates hypertension and albuminuria [53], whereas ovariectomy performed in young female NZBWF1 mice (10 weeks old) delays the production of autoantibodies and onset of albuminuria without impacting blood pressure [54]. These data suggest that estrogen has a complicated temporal role in the pathogenesis of SLE and the associated hypertension.

### Metabolic Factors

Autoimmune diseases like SLE are commonly associated with metabolic changes including insulin resistance and dyslipidemia [55]. Adipokines are pleiotropic factors secreted by white adipose tissue that may contribute to chronic low-grade inflammation in autoimmune disease [56]. Adipokines like leptin, adiponectin, visfatin, and resistin have been identified as potential mediators of metabolic syndrome and associated with insulin resistance, central obesity, dyslipidemia, and hypertension in autoimmune disease. Leptin is a known immunomodulatory hormone and has also been shown to impact blood pressure through changes in sympathetic outflow and renal sodium retention [57]. There is now emerging evidence to suggest that leptin has proinflammatory effects in multiple autoimmune disease including SLE, RA, and multiple sclerosis (MS) [58]. Hyperleptinemia in SLE patients correlates directly with disease activity and inversely with numbers of circulating T_REG_. T_REG_ are critical for maintaining immunological tolerance to prevent autoimmunity, and a deficiency in T_REG_ has been linked with the development of hypertension [59•]. The known connections to the regulation of both immune and cardiovascular function highlight the need to study leptin, and other metabolic factors, and to understand both their mechanistic importance and value as therapeutic targets in autoimmune-associated hypertension.

### Immune System Dysfunction: The Role of B and T Cells

Recent studies from our laboratory demonstrated a key mechanistic role of immune system dysfunction in hypertension associated with autoimmunity in the NZBWF1 female mouse model of SLE [10, 11, 60]. The depletion of B cells with anti-CD20 antibodies (the mouse equivalent of rituximab) [60] and mycophenolate mofetil (MMF) [11] attenuated hypertension in this mouse model. Importantly, both rituximab and MMF are used clinically in human patients with SLE [61, 62] and MMF has been shown to reduce blood pressure in small clinical studies in humans [63]. A more recent study from our laboratory addressed the question of whether autoantibodies mechanistically contribute to the pathogenesis of hypertension during autoimmune disease. In that study, Taylor et al. utilized the proteasome inhibitor bortezomib to deplete plasma cells and demonstrated the importance of circulating immunoglobulins to the pathogenesis of hypertension during autoimmunity [9•]. Bortezomib is a selective inhibitor of the 26S proteasome and induces cell death by activating the terminal unfolded protein response. Plasma cells are hypersensitive to proteasome inhibition compared to other cell types because of their extremely high rate of protein biosynthesis [64].

In humans and in experimental animal models, there is a decrease in circulating T_REG_ cells that is linked to the development of SLE [65–70]. Adoptive transfer of T_REG_ in lupus-prone mice results in delayed disease progression, reduced renal pathology, and increased survival [71]. Similarly, adoptively transferring T_REG_ into experimental models of hypertension reduces blood pressure [72–74]. These studies further strengthen the link between autoimmunity and blood pressure control. A recent study from our laboratory indirectly supports the concept that expanding T_REG_ during SLE may attenuate autoimmune-associated hypertension [10]. Treatment with an anti-CD3ε antibody, which has been utilized to induce peripheral immune tolerance and expand T_REG_ cells in small clinical studies [75], attenuated the hypertension in female SLE mice [10]. Although there are currently no studies directly testing whether expansion of the T_REG_ population attenuates hypertension during SLE, Liu et al. [76] reported that CD8+i T_REG_ attenuate LPS-induced glomerular endothelial cell injury in female MRL/lpr lupus-prone mice by inhibiting p38 MAPK and the NF-κB pathway. The role of CD8+i T_REG_ and other T cell subsets in autoimmune disease-associated hypertension remains to be examined. Interleukin-17A (IL-17A) is a proinflammatory cytokine produced predominantly by CD4+ T-helper 17 cells as well as γδ T cells that play a potentially important role in autoimmune diseases. Solak et al. recently demonstrated that IL-17A mediates AngII-induced renal injury and regulates renal sodium transporters, NHE3 and sodium-chloride cotransporter (NCC), via an SGK1-dependent pathway [77]. This study provided mechanistic insight into how inflammatory cytokines like IL-17A can regulate sodium and water homeostasis and blood pressure [77].

### Vascular Dysfunction

Numerous studies reported that patients with SLE or RA exhibit impaired vascular function as measured by endothelial dependent, flow-mediated dilatation [78, 79]. A recent study found that RA disease duration is a predictor of vascular stiffness as measured by carotid to femoral pulse wave velocity (cfPWV) [80]. Similarly, a meta-analysis from patients with primary Sjogren’s syndrome showed a significant increase in pulse wave velocity and intima media thickness suggestive of arterial stiffness and subclinical atherosclerosis [81]. In SLE, circulating autoantibodies and other inflammatory mediators can activate endothelial cells to express cell adhesion molecules [82, 83]. Endothelial cell adhesion molecules (ECAMS), such as vascular cellular adhesion molecule-1 (VCAM-1) and intracellular adhesion molecule-1 (ICAM-1), are transmembrane proteins that may be important mediators in the migrations of immune cells from the circulation to tissues affected by inflammation [84]. Increased serum and urine levels of VCAM-1 and ICAM-1 have been found in patients with active SLE [85–87], and ECAMs are a typical finding on the histopathology of glomerular lesions in MRL/lpr mice [88]. Overall, the infiltration of immune cells and impact of inflammatory cytokines on vascular function during SLE as it related to the development of hypertension during autoimmunity is an open area of investigation. It is well known that vascular dysfunction is associated with the development of hypertension and that renal vascular dysfunction in particular can directly cause hypertension.

### Renal Hemodynamics

Body salt and fluid homeostasis and are essential for normal blood pressure control, and renal hemodynamic changes that lead to increased sodium and water reabsorption and extracellular fluid volume underlie the development of hypertension. Renal involvement in SLE typically occurs in 50–75% of patients in form of immune complex mediated glomerulonephritis [89]. The nephritis often results from extra-glomerular immune deposits found in the tubular basement membranes, the interstitium, and blood vessels [90]. The renal injury per se that is caused by nephritis is likely not the cause of the hypertension. This is supported by studies showing that hypertension is prevalent in child-onset SLE independent of the presence of nephritis [91]. In addition, Shaharir et al. found that 53% of SLE patients were hypertensive without evidence of nephritis [20]. Consistent with human data, our laboratory recently reported that hypertension is not associated with markers of renal injury, including albuminuria and glomerulosclerosis in the NZBWF1 female mouse model of SLE [9•]. Taken together, both clinical data and data from experimental animal models suggest the possibility that early immunological changes during autoimmunity may impact renal hemodynamic and tubular function to cause hypertension before there are clear signs of renal injury.

### Alterations in Glomerular Filtration Rate and Renal Blood Flow

Surprisingly, little is known about how the immunological changes that occur in patients with autoimmune disease directly impact renal hemodynamic function and increase the risk of developing hypertension. Clinical data show impairments in both GFR and renal plasma flow (measured using urinary clearance methods) in SLE patients with active and inactive lupus nephritis [92]. Although few studies have focused on renal hemodynamic changes in SLE in recent years, Mori et al. found that out of 1908 patients with RA identified in Japanese community hospitals, 33.8% had renal dysfunction with absolute eGFR < 60 ml/min, and 2.1% of patients had severe or end-stage kidney disease with absolute eGFR < 30 ml/min [93]. Of the patients diagnosed with renal dysfunction, 60.1% were classified as hypertensive (systolic blood pressure ≥ 140 mmHg) compared to 26.3% without renal dysfunction [93]. Another study reported that the 20-year cumulative incidence of reduced kidney function was significantly higher in RA patients compared to non-RA patients [94]. Evidence that renal inflammation is a key element in the role of the kidney in the pathogenesis of hypertension has been reviewed recently by Bernardo Rodriguez-Iturbe [13]. A study by Franco et al. found that AngII hypertension was associated with renal inflammation with a high salt diet and resulted in increased afferent and efferent glomerular vasoconstriction and a reduction in renal plasma flow and single nephron GFR [95]. MMF treatment offered protection in the AngII-salt-sensitive hypertensive rat model by correcting glomerular hemodynamics in association with immunosuppression [95]. Still, the specific immunological mediators that alter renal function during autoimmune diseases and the timing of the changes have not been clearly elucidated.

### Renin-Angiotensin System

The renin-angiotensin system (RAS) is widely recognized for its role in blood pressure control and is also recognized for its role as a proinflammatory mediator. Therefore, RAS blockade in patients with autoimmune disease may have dual effects in managing autoimmune disease and its associated hypertension. A study from our laboratory showed the renal vascular sensitivity to AngII is enhanced in female NZBWF1 mice with SLE suggesting a direct impact of autoimmunity on AngII-mediated renal hemodynamic function [96]. In a more recent study, Yue et al. found that early (within 3 months after kidney biopsy) blockade of the RAS in SLE patients with antiphospholipid-associated nephropathy (aPLN) improved both short- (12 months after kidney biopsy) and long-term (a mean follow-up of 80 months) renal outcomes like improvement ratio of eGFR [97]. Interestingly, blood pressure was not adequately controlled by RAS blockade in this patient population and the renal improvements occurred independently of changes in blood pressure or albuminuria. Thus, despite the usage of RAS blockade as an initiator for blood pressure control during SLE, a large knowledge gap remains for understanding the best clinical practices for treating hypertension in these patients.

### Oxidative Stress

Reactive oxygen species (ROS), such as superoxide anion, hydrogen peroxide, and hydroxyl anion, are known to play a major role in the tissue damage associated with autoimmune disease and in the development of hypertension [98, 99]. The impact of ROS on renal function and the development of hypertension have been extensively reviewed [15]; however, less is understood about the impact of ROS on autoimmune-associated hypertension.

### Neutrophils

Neutrophils are specialized phagocytic cells of the innate immune system that play a key role in the release of ROS during an autoimmune inflammatory response [100]. The main source of ROS in neutrophils is NADPH oxidase 2 (NOX2); however, mitochondrial ROS are likely a major source of tissue damage resulting from activation of the inflammasome and neutrophil extracellular trap (NET) formation [100]. NETosis is a cell death pathway, similar to apoptosis, characterized by extrusion of chromatin bound to cytosolic and granular content, and is a normal host defense mechanism designed to trap and kill microorganisms. NETs and NETing neutrophils are found in the kidneys of patients with SLE, where they can be a source of nuclear antigens [101]. Low-density granulocytes (LDGs) present in peripheral blood mononuclear cell (PBMC) fractions of lupus patients reportedly have a propensity to form NETs, which promote vascular leakage and endothelial dysfunction through the degradation of vascular endothelial cadherin and subsequent activation of β-cadherin signaling [102, 103•]. Another study reported a correlation between renal involvement in SLE and impaired NET degradation that is associated with altered DNase1 function [104]. Whether impaired degradation of NETs represents an important early signal for the development of autoantibodies that can promote hypertension, or directly increases oxidative stress in the renal vasculature and tubules to promote hypertension has not been addressed experimentally.

### Mitochondrial Dysfunction

Apoptosis and NETosis are both energy-consuming processes that require mitochondria as an energy source, and mitochondria are a major cellular source of intracellular ROS generation [105]. Growing evidence suggests a role for mitochondrial dysfunction and the subsequent generation of ROS in diabetic nephropathy and chronic kidney disease [106]; however, very little is understood about renal mitochondrial dysfunction during SLE and its potential role in the pathogenesis of hypertension. T cells from patients with SLE are reported to exhibit mitochondrial hyperpolarization, increased ROS production, diminished intracellular glutathione (GSH) levels, cytoplasmic alkalization, and adenosine triphosphate (ATP) depletion that causes diminished activation-induced apoptosis and sensitizes lupus T cells to necrosis [107]. Given the role of T cell subset in SLE disease progression, and evidence pointing to a role for T cells in the development of hypertension, it will ultimately be important to understand the impact of mitochondrial dysfunction in these and other immune cells on physiological systems that ultimately control blood pressure.

## Conclusions

The management of high blood pressure in patients with autoimmune disease should be considered a major aspect of their treatment plan. In this review, we have provided an update on some of the disease-specific factors that contribute to the prevalent hypertension of autoimmunity. Recent studies from our laboratory have highlighted the important role of the adaptive immune system in the development of hypertension in an experimental mouse model of SLE. Future studies should explore the impact of immune cell infiltration in the kidney to promote changes in renal hemodynamics. Neutrophils, impaired NET degradation, and mitochondrial dysfunction may lead to the formation of auto-antigens and the production of ROS that could promote changes in glomerular filtration rate, renal vascular resistance, and renal blood flow, ultimately leading to hypertension, and an increased risk for cardiovascular and renal disease.
